# Cell-Free Mitochondrial DNA in Acute Brain Injury

**DOI:** 10.1089/neur.2022.0032

**Published:** 2022-09-28

**Authors:** Saeed Kayhanian, Angelos Glynos, Richard Mair, Andras Lakatos, Peter J.A. Hutchinson, Adel E. Helmy, Patrick F. Chinnery

**Affiliations:** ^1^ Department of Clinical Neurosciences, University of Cambridge, Cambridge, United Kingdom.; ^2^ MRC Mitochondrial Biology Unit, University of Cambridge, Cambridge, United Kingdom.; ^3^ Department of Neurosurgery, Cambridge University Hospitals, Cambridge, United Kingdom.; ^4^ Department of Neurology, Cambridge University Hospitals, Cambridge, United Kingdom.

**Keywords:** acute brain injury, brain inflammation, DAMP, mitochondrial DNA, subarachnoid hemorrhage, traumatic brain injury

## Abstract

Traumatic brain injury and aneurysmal subarachnoid haemorrhage are a major cause of morbidity and mortality worldwide. Treatment options remain limited and are hampered by our understanding of the cellular and molecular mechanisms, including the inflammatory response observed in the brain. Mitochondrial DNA (mtDNA) has been shown to activate an innate inflammatory response by acting as a damage-associated molecular pattern (DAMP). Here, we show raised circulating cell-free (ccf) mtDNA levels in both cerebrospinal fluid (CSF) and serum within 48 h of brain injury. CSF ccf-mtDNA levels correlated with clinical severity and the interleukin-6 cytokine response. These findings support the use of ccf-mtDNA as a biomarker after acute brain injury linked to the inflammatory disease mechanism.

## Introduction

Acute brain injury, encompassing both traumatic brain injury (TBI) and aneurysmal subarachnoid hemorrhage (aSAH), is a leading cause of death and disability. There are estimated to be ∼50 million cases of TBI and 5 million cases of aSAH (accounting for ∼5% of all strokes) occurring annually worldwide.^1,2^ Although our understanding of the mechanisms of cellular injury and death after physical and ischaemic insult remains incomplete, mitochondrial dysfunction has been implicated in the propagation of secondary injury through oxidative stress, calcium dysregulation, and by activating apoptosis.^3^

Mitochondria contain their own genome (mitochondrial DNA; mtDNA), which codes for 13 essential proteins involved in oxidative energy metabolism. When released from organelles and cells, mtDNA contributes to damage-associated molecular patterns (DAMPs), and raised plasma levels of circulating cell-free mtDNA (ccf-mtDNA) are associated with organ dysfunction after systemic trauma.^4,5^ An early neuroinflammatory response is evident after acute brain injury, demonstrated by the release of cytokine mediators, such as Interleukin-6 (IL-6), within hours of injury.^6^ However, the links between serum and cerebrospinal fluid (CSF) levels of ccf-mtDNA after acute brain injury, and their relationship to an early inflammatory response, have not been systematically examined. We hypothesized that levels of ccf-mtDNA in both CSF and serum would be raised after acute brain injury and correlate with the neuroinflammatory response.

## Methods

### Subjects

Patients with severe TBI or aSAH requiring admission to the Neuro-Critical Care Unit at Cambridge University Hospitals (CUH) were recruited within 48 h of injury. Serum was extracted from blood samples taken from an arterial line. Next, 5 mL of CSF was extracted as a fresh, clean sample from the external ventricular drain when present. All samples of serum and CSF (when available) were obtained within 48 h of injury. This study was approved by the NHS Health Research Authority and the CUH institutional ethics board.

Control CSF and serum samples were obtained from patients undergoing investigation for normal pressure hydrocephalus or idiopathic intracranial hypertension. These patients underwent lumbar drain or lumbar infusion studies, requiring a lumbar puncture and removal of CSF as part of standard clinical investigations. All control patients consented to use of their CSF and serum for research purposes as part of the CUH Neurosurgical Biobank.

All CSF samples were centrifuged within 15 min of collection at 2000*g* for 10 min at 4°C with the supernatant stored in cryovials at −80 ^o^C. Blood samples were collected into tubes (serum gel, 3.9 mL; Sarstedt AG & Co. KG, Nümbrecht, Germany), gently inverted three times and left to clot for 30 min. Tubes were then centrifuged at 2000g for 10 min at 4°C. The supernatant was then stored at −80°C.

Demographic and clinical data were extracted from the electronic health record system, with the initial Glasgow Coma Scale (GCS) as the best responses at the point of first presentation to a clinician.

### Mitochondrial DNA copy number and interleukin-6 measurements

Quantification of ccf-mtDNA was performed in triplicate by droplet digital polymerase chain reaction amplification of the mitochondrial genes, *MTND1* and *MTND4*, using the Bio-Rad QX100 Digital Droplet PCR platform (Bio-Rad Laboratories, Hercules, CA). For serum samples, DNA was first extracted using the DNeasy Blood and Tissue kit (Qiagen, Hilden, Germany). CSF samples were loaded directly, without DNA extraction. The absolute mtDNA copy number is expressed in copies per microliter, derived as the mean of ND1 and ND4 values. Cases and control samples were randomly assigned to each run. IL-6 concentration was measured in duplicate, directly from both serum and CSF samples, using the Human IL-6 ELISA kit (Abcam, Cambridge, UK).

### Statistical analysis

Data were analyzed using GraphPad Prism (Version 9; GraphPad Software, La Jolla, CA) and R statistical language software (R Foundation for Statistical Computing, Vienna, Austria). Statistical significance testing was carried out using data appropriate tests (detailed in the text), with significance level set at *p* < 0.05.

## Results

Twenty-five acute brain injury (15 TBI and 10 aSAH) and 10 control patients were recruited. Mean age (±SD [standard deviation]) in the cohorts was 52.6 (±16.8), 64.8 (±9.3), and 66.9 (±14.6) years for TBI, aSAH, and control groups, respectively (Kruskal-Wallis' test, *p* = 0.067). Median initial GCS score was lower in the TBI compared with aSAH cohort (6 vs. 10, respectively), which did not reach significance (Mann-Whitney's U test, *p* = 0.16). Demographic data for the cohort are summarized in Table 1.

**Table 1.** Demographic and Clinical Features of the Participants

	TBI (*n* = 15)	aSAH (*n* = 10)	Control (*n* = 10)	*p* value
Age (years), mean ± SD	52.6 ± 16.8	64.8 ± 9.3	66.9 ± 14.6	0.067
Male [%]	9 [60]	4 [40]	8 [80]	**0.036**
Admission GCS, median [range]	6 [4–14]	10 [4–14]	N/A	0.071
Motor score, median [range]	3 [1–6]	5 [2–6]	N/A	0.1
Injury Severity Score, median [range]	21 [16–29]	N/A	N/A	N/A

TBI, traumatic brain injury; aSAH, aneurysmal subarachnoid hemorrhage; SD, standard deviation; GCS, Glasgow Coma Scale; N/A, not applicable.

Serum samples were collected from all brain injury and control patients. CSF was available and collected from all aSAH patients, 5 TBI, and all control patients. Median time of sampling (± interquartile range) was 24 (±26) and 26 h (±19) after injury for the TBI and aSAH cohorts, respectively (Mann-Whitney's U test, *p* = 0.88).

### Acute brain injury patients demonstrate raised circulating cell-free mitochondrial DNA levels in both cerebrospinal fluid and serum

**FIG. 1.**
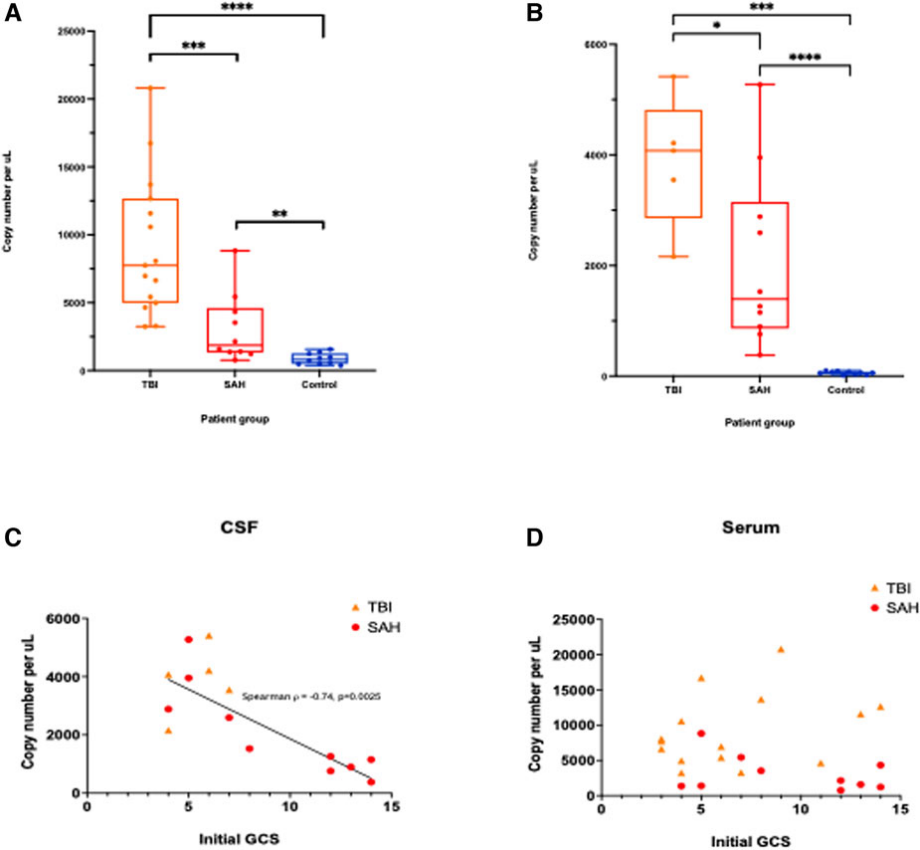
(**A**) Serum levels of ccf-mtDNA copy number by patient group. Asterisks indicate statistical significance: ****p < 10^–4^, ***p < 10^–3^, **p < 10^–2^. (**B**) CSF levels of ccf-mtDNA copy number by patient group. Asterisks indicate statistical significance, ****p < 10^–4^, ***p < 10^–3^, *p < 10^–1^. (**C**) Correlation between initial GCS and ccf-mtDNA copy number in CSF. Spearman: ρ = −0.74, *p* = 0.0025. (**D**) Correlation between initial GCS and ccf-mtDNA copy number in serum. Spearman: ρ = −0.17, *p* = 0.43. ccf-mtDNA, circulating cell-free mitochondrial DNA; CSF, cerebrospinal fluid; GCS, Glasgow Coma Scale; SAH, subarachnoid hemorrhage; TBI, traumatic brain injury.

In the TBI cohort, ccf-mtDNA copy number was significantly raised compared to controls in both serum (Mann-Whitney's U test, *p* < 0.0001) and CSF (*p* = 0.0007). Similarly, in the aSAH cohort, ccf-mtDNA copy number was raised compared to controls in both serum (*p* = 0.0015) and CSF (*p* < 0.0001). Between the two acute brain injury cohorts, there was a higher ccf-mtDNA mean copy number in TBI compared with SAH in both serum (*p* = 0.0009) and CSF (*p* = 0.04; Fig. 1A,B).

### Circulating cell-free mitochondrial DNA copy number in cerebrospinal fluid correlates to the severity of brain injury

Initial GCS score correlated with ccf-mtDNA copy number in CSF (Spearman, ρ = −0.74, *p* = 0.0025), but not serum (Spearman, ρ = −0.17, *p* = 0.43; Fig. 1C,D). There was no correlation between ccf-mtDNA and Injury Severity Score for TBI patients, in either CSF (Spearman, ρ = −0.20, *p* = 0.78) or serum (Spearman, ρ = −0.25, *p* = 0.35).

### Interleukin-6 concentration in cerebrospinal fluid correlates with circulating cell-free mitochondrial DNA level

**FIG. 2.**
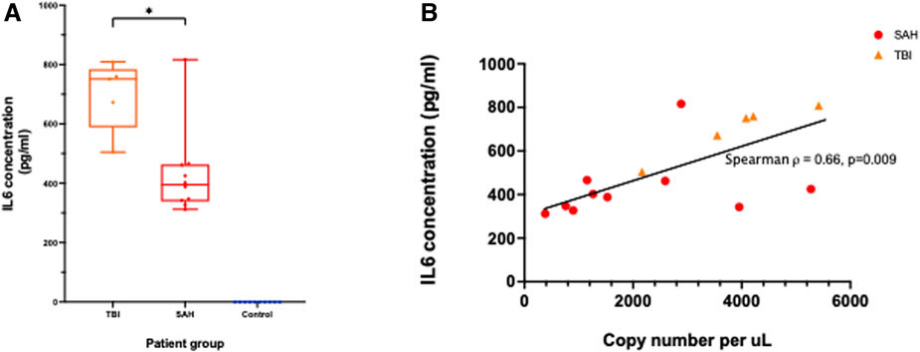
(**A**) CSF concentrations of IL-6 by patient group. Asterisks indicate statistical significance: *p < 10^–1^. (**B**) Correlation of ccf-mtDNA copy number with the concentration of IL-6 in the CSF. ccf-mtDNA, circulating cell-free mitochondrial DNA; CSF, cerebrospinal fluid; IL-6, interleukin-6; SAH, subarachnoid hemorrhage; TBI, traumatic brain injury.

There was no detectable IL-6 in either serum or CSF of control patients. Mean concentration of IL-6 in CSF was raised compared to controls for both the TBI (699.3 pg/mL; Mann-Whitney's U test, *p* = 0.0003) and aSAH (428.9 pg/mL; *p* < 0.0001) cohorts (Fig. 2A). Mean serum concentrations (±SD) of IL-6 were comparably low for TBI (36.9 ± 44.4 pg/mL) and SAH (6.0 ± 9.0 pg/mL) cohorts (Mann-Whitney's U test, *p* = 0.08). CSF concentration of IL-6 correlated with CSF ccf-mtDNA copy number (Spearman, ρ = 0.66, *p* = 0.009; Fig. 2B).

## Discussion

We demonstrate that ccf-mtDNA is raised in both serum and CSF after acute brain injury, and that CSF levels correlate with both severity of injury and an inflammatory cytokine response. This presents ccf-mtDNA as a plausible biomarker of acute brain injury, with potential important implications for our mechanistic understanding of the role of mitochondria in acute brain injury and neuroinflammation.

It is notable that, although both serum and CSF ccf-mtDNA levels are higher in TBI compared to aSAH, it is only CSF levels that correlate with the initial GCS. This correlation has also been shown in a cohort of pediatric TBI patients.^7^ Given that there is considered to be a more widespread burden of cellular injury in TBI compared to aSAH, and with the assumption that CSF offers more of a “real-time” and local reflection of the milieu of the brain compared to serum, these results together suggest that CSF ccf-mtDNA levels reflect the burden of central nervous system injury. Further investigation is necessary to explore whether this is an active process where mtDNA is released from intact cells or a passive reflection of cell death.

In either scenario, the presence of raised ccf-mtDNA in the central nervous system in the context of injury is of interest. mtDNA, unlike nuclear DNA, contains unmethylated CpG sequences—a pattern common to bacterial DNA—which act as a DAMP.^8,9^ These stimulate an innate immune response and proinflammatory cytokine release through a variety pattern recognition receptors, for instance Toll-like receptor 9 and cyclic GMP-AMP synthase, which are known to be expressed in neurons and glia.^10–12^ Further, IL-6 has been specifically implicated as downstream to both intra- and extracellular mtDNA-induced inflammatory response, including in the context of a genetic variant of Parkinson's disease (PRKN-PINK) characterized by mitochondrial dysfunction and impaired mitophagy.^13,14^

After acute brain injury, there is ample evidence of a significant neuroinflammatory response, with IL-6 implicated as a prototypical cytokine released within hours of injury.^15^ However, the mechanism for this response is poorly understood, and this is highlighted by the failure of numerous clinical trials for broad “anti-inflammatory” agents (e.g., corticosteroids) in these cohorts.^15^ In concordance with previous studies, we demonstrate an acute IL-6 cytokine response in brain injury patients that is isolated to the CSF and reflects the severity of injury.^6^ We found that this response is highly correlated to the ccf-mtDNA copy number in CSF. This finding, alongside existing *in vitro* evidence of mtDNA inducing IL-6 production, suggests a role for ccf-mtDNA as an initiator of neuroinflammation after acute brain injury.^13,14^ mtDNA thus presents as both a potential biomarker for the process of neuroinflammation and a target for investigating how this process is initiated. mtDNA, or its specific downstream inflammatory effectors, may also present new avenues for therapeutic intervention, as has already been explored pre-clinically, using nucleic acid scavenging polymers.^5^

One limitation of this study is the relatively small sample size, which was insufficiently powered to stratify patients by demographic parameters, such as age or sex, or pre-morbid conditions. As such, we have not attempted to draw any conclusions with regard to prognostication or association to long-term outcome, but these warrant future exploration in larger longitudinal studies, with more frequent sampling, alongside further measures of injury severity such as imaging scores. We highlight also that our control cohort was drawn from patient groups undergoing investigation for neurological diseases, but we found that both serum and CSF copy number values of ccf-mtDNA of control subjects in this study resemble previous healthy subject findings.^16,17^

In summary, we demonstrate ccf-mtDNA as a potential molecular marker of acute brain injury with CSF levels reflecting severity of brain injury and correlating with an inflammatory cytokine response. These findings open a new avenue for investigating the mechanism and treatment of neuroinflammation after acute brain insults.

## Supplementary Material

Supplemental data

## Abbreviations Used

aSAH: aneurysmal subarachnoid hemorrhage
ccf-mtDNA: circulating cell-free mtDNA
CSF: cerebrospinal fluid
CUH: Cambridge University Hospitals
DAMPs: damage-associated molecular patterns
GCS: Glasgow Coma Scale
IL-6: interleukin-6
mtDNA: mitochondrial DNA
SD: standard deviation
TBI: traumatic brain injury
