# Supplementary material for: Cell-Free Mitochondrial DNA in Acute Brain Injury
Source: Neurotrauma Rep. 2022 Sep 28;3(1):415–20. doi: 10.1089/neur.2022.0032 (PMC9531878; doi:10.1089/neur.2022.0032)

**Supplementary Figure 1** Correlation between Injury Severity Score and ccf-mtDNA copy number in the TBI cohort (A) serum, Spearman ρ=-0.25, p=0.35 and (B) CSF, Spearman ρ=-0.20, p=0.78.


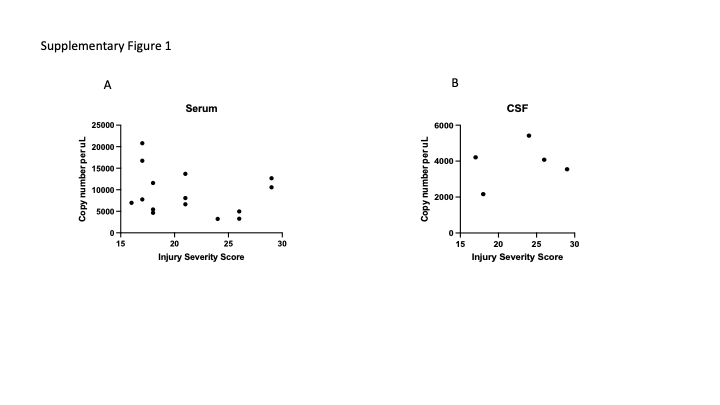

Supplement: Supplemental data [file Supp_FigS1.docx]
